# Fucosidosis: A Review of a Rare Disease

**DOI:** 10.3390/ijms26010353

**Published:** 2025-01-03

**Authors:** Burcu Pekdemir, Mikhael Bechelany, Sercan Karav

**Affiliations:** 1 Department of Molecular Biology and Genetics, Çanakkale Onsekiz Mart University, Çanakkale 17100, Turkey; burcupekdemir0@gmail.com; 2 Institut Européen des Membranes (IEM), UMR 5635, University Montpellier, ENSCM, CNRS, F-34095 Montpellier, France; 3 Functional Materials Group, Gulf University for Science and Technology (GUST), Masjid Al Aqsa Street, Mubarak Al-Abdullah 32093, Kuwait

**Keywords:** α-L-fucosidase, *FUCA1*, autosomal recessive, lysosomal storage disorders, rare disease, enzyme deficiency

## Abstract

Fucosidosis is a rare lysosomal storage disease caused by α-L-fucosidase deficiency following a mutation in the *FUCA1* gene. This enzyme is responsible for breaking down fucose-containing glycoproteins, glycolipids, and oligosaccharides within the lysosome. Mutations in *FUCA1* result in either reduced enzyme activity or complete loss of function, leading to the accumulation of fucose-rich substrates in lysosomes. Lysosomes become engorged with undigested substrates, which leads to secondary storage defects affecting other metabolic pathways. The central nervous system is particularly vulnerable, with lysosomal dysfunction causing microglial activation, inflammation, and neuronal loss, leading to the neurodegenerative symptoms of fucosidosis. Neuroinflammation contributes to secondary damage, including neuronal apoptosis, axonal degeneration, and synaptic dysfunction, exacerbating the disease process. Chronic neuroinflammation impairs synaptic plasticity and neuronal survival, leading to progressive intellectual disability, learning difficulties, and loss of previously acquired skills. Inflammatory cytokines and lysosomal burden in motor neurons and associated pathways contribute to ataxia, spasticity, and hypotonia, which are common motor symptoms in fucosidosis. Elevated neuroinflammatory markers can increase neuronal excitability, leading to the frequent occurrence of epilepsy in affected individuals. So, fucosidosis is characterized by rapid mental and motor loss, along with growth retardation, coarse facial features, hepatosplenomegaly, telangiectasis or angiokeratomas, epilepsy, inguinal hernia, and dysostosis multiplex. Patients usually die at an early age. Treatment of fucosidosis is a great challenge, and there is currently no definitive effective treatment. Hematopoietic cell transplantation studies are ongoing in the treatment of fucosidosis. However, early diagnosis of this disease and treatment can be effective. In addition, the body’s immune system decreases due to chemotherapy applied after transplantation, leaving the body vulnerable to microbes and infections, and the risk of death is high with this treatment. In another treatment method, gene therapy, the use of retroviral vectors, is promising due to their easy integration, high cell efficiency, and safety. In another treatment approach, enzyme replacement therapy, preclinical studies are ongoing for fucosidosis, but the blood–brain barrier is a major obstacle in lysosomal storage diseases affecting the central nervous system. Early diagnosis is important in fucosidosis, a rare disease, due to the delay in the diagnosis of patients identified so far and the rapid progression of the disease. In addition, enzyme replacement therapy, which carries fewer risks, is promising.

## 1. Introduction

**Figure 1:**
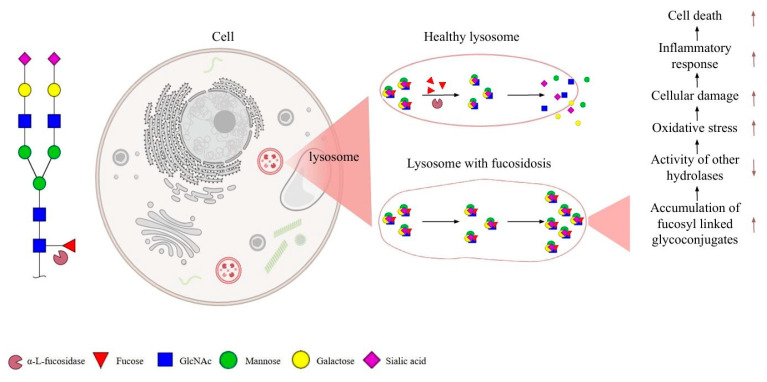
Fucosidosis is a lysosomal storage disease characterized by a deficiency of α-L-fucosidase. Fucose-linked conjugates accumulated in lysosomes can inhibit the activity of hydrolases, and the dysfunction of lysosomes leads to cell death. Upward brown arrows represent an increase in the processes, whereas downward brown arrows indicate a decrease (created with Biorender).

α-L-fucosidase deficiency is observed after a mutation in the *FUCA1* gene. Deficiency of this enzyme causes the accumulation of α-L-fucose-linked glycoconjugates in tissue lysosomes of different organs [1]. Fucosidosis is a lysosomal storage disease (LSD) characterized by the accumulation of these sugars in α-L-fucosidase deficiency. This autosomal recessive and rare disease negatively affects many other systems, especially the central nervous system. In particular, it appears to cause rapid loss of mental and motor functions, causing irreversible damage [2]. Fucose-linked conjugates accumulated in lysosomes can inhibit the activity of hydrolases by changing the substrate-binding properties of other hydrolases or by affecting the stability of enzymes [3]. This situation of the activity of other hydrolases in lysosomes prevents the separation of fucose-linked glycoconjugates into monomers and causes their storage. Studies examining the hydrolysis of glycoproteins showed that fucose must first be removed for the activity of the *N*-glycanase aspartylglucosaminidase, which hydrolyzes the bond between *N*-linked glycans and peptides [4]. However, substrate accumulation is observed in α-L-fucosidase deficiency and lysosomal dysfunction leads to necrosis and apoptosis of cells (Figure 1) [5].

### History of the Disease

The first case of fucosidosis was seen in a study conducted by Durand et al. in 1966. In this case, seen in two siblings, the clinical picture of the 3-year study reported gradually increasing memory loss, mental disorders, and degeneration in motor functions. As a result of histochemical studies, they observed the accumulation of a material consisting of a mixture of mucopolysaccharides and lipids in various organs and tissues of the body, including the central nervous system. Along with the abnormal mitochondria, the liver biopsy result showed a swollen appearance in balloon or pseudogargoyle cells. Although an idea was obtained about the composition of the accumulated material as well as the clinical picture obtained in this study, its definitive chemical definition could not be made with the histochemical techniques available at that time. It was also seen to be different from other lysosomal storage diseases [6].

In another study conducted by Van Hoof and Hers in 1968, the activity of enzymes found in different tissue lysosomes of patients with Hurler syndrome or related diseases was analyzed. The results showed that the α-L-fucosidase enzyme showed an excessive increase in lysosomes in two out of five patients, but this enzyme was not active in the brains and livers of three patients. At the same time, attention was drawn to the significant increase in the amount of mucopolysaccharides seen in Hurler syndrome in the tissues of these three patients. Later, mucopolysaccharides were isolated from the livers of these three patients, and their molecular structures were examined by chromatographic analysis. They reported that there was a large accumulation of fucose derivatives in the structures of these monosaccharides. The clinical report of these patients was given by Durand and colleagues, who had previously reported the accumulation of two unusual glycolipids in the liver. In the first study conducted by Durand and colleagues, the deficiency of the α-L-fucosidase enzyme was thus identified retrospectively. Van Hoof and Hers later proposed to name this new lysosomal storage disease mucopolysaccharidosis F (F for fucosidosis) because of the similar histological findings and symptoms seen in Hurler syndrome [7]. Later, Durand and his colleagues continued their studies on this case and confirmed that the substances accumulated in the tissues were due to the deficiency of α-L-fucosidase and that fucose accumulated in all tissues in its pathogenesis. They proposed the term ‘fucosidosis’ for the inborn error of carbohydrate metabolism and included sphingolipids and polysaccharides containing fucose under this name. Thus, a new lysosomal storage disease continued to be named in this way in the literature [8].

Later, Kousseff et al. stated that there may be two different types of fucosidosis. Although α-L-fucosidase activity is deficient in both types, rapidly progressive psychomotor and neurological deterioration was observed in type I fucosidosis. It was also stated that patients died in early childhood (before approximately 5 years of age). In type II fucosidosis, psychomotor retardation and neurological deterioration were observed that progressed more slowly than in type I. In this type, where longer survival was observed, evidence of lysosomal storage was also found in angiokeratomas, vascular endothelium, and skin fibroblasts [9].

In addition to the description of 58 patients in the literature in 1989 from 105 previously published reports and international survey studies, Willems et al. prepared a review in 1991 by examining a total of 77 patients, 19 of which had not been previously reported. The age of death was recorded in 44 patients in the review report. The number of patients who died before the age of 10 was recorded as 19 (43%), while the number of patients who died after the age of 20 was recorded as 18 (41%). The number of patients who died between the ages of 10 and 20 was determined as seven (16%), indicating a bimodal distribution. It was suggested that the clinical variability among the patients was determined by at least four different mutations, but the description of more fucosidosis patients caused clinical gaps between type I and type II. It was later thought that this clinical variability was not only due to different allelic mutations in *FUCA1*, but additional factors also contributed to the phenotype of the fucosidosis patient. According to the distribution and incidence report of the disease presented by the same review, since only 77 patients were identified in a comprehensive screening in North America, Western Europe, Japan, and Australia, the incidence of fucosidosis was determined to be extremely low. While Italians and the Mexican–Indian population in New Mexico and Colorado in the USA showed a high incidence, fucosidosis disease was not detected only in the Australian continent. The high incidence of fucosidosis patients (20), the majority of whom were of Italian origin, was explained by the high consanguineous marriage and founder effect found in the Italian population [2]. Fucosidosis is a rare disease with an estimated incidence of less than 1 in 200,000 live births worldwide, and recent studies of the disease have identified a high incidence in Cuba [10,11,12].

There is no definitive cure for fucosidosis, and treatments aimed at reducing clinical symptoms are being applied [13]. However, studies on hematopoietic stem cell transplantation, gene therapy, and enzyme replacement therapy are ongoing. But, the accessibility and feasibility of these advanced therapies for rare diseases, like fucosidosis, are significantly influenced by socioeconomic factors. Socioeconomic inequalities may also affect early diagnosis and initiation of treatment, as low-income families may face barriers such as delayed access to diagnostic tools, limited awareness, and logistical difficulties in reaching treatment centers. Diagnostic delays cause these treatment strategies to be ineffective.

## 2. Other Lysosomal Storage Diseases

Lysosomal storage diseases are characterized by the accumulation of macromolecules in lysosomes as a result of a deficiency of lysosomal hydrolases. In these diseases, which are generally inherited as autosomal recessive, sphingolipids, and mucopolysaccharides, glycolipids and oligosaccharides accumulate in different tissues and lysosomes of the body, resulting in excessive activation of the inflammatory response and cell death. Lysosomal storage diseases are categorized according to the substance that cannot be hydrolyzed and stored as a result of enzyme deficiency [14]. For example, in the glycogen storage disease type II known as Pompe disease, carbohydrates accumulate in the glycogen structure in cell lysosomes as a result of lysosomal acid α-glycosidase deficiency. Cholesteryl ester storage disease and Wolman disease are characterized by the storage of neutral lipids, such as cholesteryl ester, and triglycerides in lysosomes as a result of acid cholesteryl hydrolase deficiency. Sphingolipidoses known as Fabry, Tay–Sachs, Niemann-Pick, Krabbe, and Gaucher diseases, and mucolipidoses, known as Pseudo-Hurler Polydystrophy disease, are disease groups in which glycolipids are stored due to lysosomal enzyme deficiencies. Mannosidosis, aspartoglycosidosis, galactosylceramidosis, and Schindler disease are included in the glycoproteinosis group and are characterized by the storage of *N*-glycans in lysosomes as a result of the deficiency of the enzyme that breaks down glycans. Fucosidosis diseases, such as α- and β-mannosidosis, sialidosis, and aspartylglucosaminuria diseases, are diseases named according to non-hydrolyzed sugar and in which both glycolipids and glycoproteins are stored. Fucosidosis leads to the accumulation of fucose-containing glycoproteins, glycolipids, and oligosaccharides in lysosomes. The substrates that accumulate in fucosidosis contain terminal fucose residues, which are unique to this disease. This is distinct from other lysosomal storage diseases, where different enzymes and substrates are involved [15].

In lysosomal storage diseases, progressive neurological deterioration together with delayed motor and cognitive activities, seizures, and visual disturbances are common symptoms. These symptoms appear in late infancy. Gene therapy, hematopoietic stem cell transplantation, and enzyme replacement therapy are clinically applicable for only a small portion of these disease groups [16]. After the initial discovery of fucosidosis, clinical observations have been confused with other lysosomal storage diseases. Fucosidosis is characterized by progressive neurodegeneration, a feature common to many lysosomal storage diseases, such as Tay–Sachs disease and metachromatic leukodystrophy [17,18]. Affected individuals often experience developmental delays, intellectual disability, seizures, and motor dysfunction. These neurological symptoms are accompanied by coarse facial features and skeletal abnormalities (dysmorphism), which resemble those seen in Hurler syndrome [19]. In addition to neurological symptoms, fucosidosis leads to coarse facial features, hepatosplenomegaly, and skeletal abnormalities caused by the accumulation of substrates in connective tissues and organs. While these characteristics are similar to mucopolysaccharidoses, joint stiffness and corneal clouding (typical of mucopolysaccharidosis) are less pronounced in fucosidosis [1]. The severe form of fucosidosis (type I) progresses rapidly and often results in death in early childhood. This clinical course is comparable to diseases, like Krabbe disease and the infantile forms of Niemann–Pick disease and Gaucher disease, which also involve early and aggressive neurodegeneration [20,21,22]. Angiokeratoma, which is among the symptoms of fucosidosis, is seen in GM1 gangliosidosis, sialidosis type II, Schindler neuroaxonal dystrophy syndrome, Kanzaki disease, galactosialidosis, and β-mannosidosis, but it is not distinctive for Fabry disease. For this reason, advanced biochemical and other molecular analyses were needed for a complete diagnosis of fucosidosis disease [6,15].

## 3. Symptoms and Clinical Features

In α-L-fucosidase deficiency, the inability to degrade fucosylated glycolipids and glycoproteins causes many clinical symptoms with the accumulation of these glycoconjugates in various tissues and organs such as the brain, liver, bone, and skin. The occurrence of significant neurodegeneration in the course of the disease leads to significant deterioration in the mental and physical functions of the patients. In addition to these, growth retardation, coarse facial features, hepatosplenomegaly, telangiectasis or angiokeratomas, epilepsy, recurrent respiratory tract infections, inguinal hernia, and dysostosis multiplex are observed (Table 1). These clinical features of fucosidosis are similar to the features of other lysosomal storage disorders, such as mucopolysaccharidosis and mucolipidosis [23]. The majority of patients with fucosidosis die at an early age due to neurological deterioration or respiratory tract infections [1].

### 3.1. Motor and Mental Function

Fucosidosis causes serious deterioration in mental and motor functions. In addition to neurological disorders, such as difficulty in standing, loss of walking ability or frequent falls, equinovarus deformity in the feet, difficulty sitting, and deterioration in language skills, patients are also described with flexion contractures and spastic quadraparesis with increased tendon reflexes. However, in rare cases, permanent incontinence, epileptic seizures, or asymmetric mild–moderate sensorineural hearing loss and eustachian tube dysfunction can be observed [1,24,25].

In the neuroimaging results of the patients, changes in white matter and gray matter were identified. In particular, the prominent white matter abnormalities of the globus pallidus in fucosidosis are considered to be a specific finding for fucosidosis, helping to distinguish it from other neurometabolic disorders [30]. The globi pallidi and substantia nigra have low signal intensity on T2/FLAIR (Fluid-Attenuated Inversion Recovery) sequences but high signal intensity on T1 sequences [1,31,32,33].

### 3.2. Facial and Physical Features

In fucosidosis, a coarse facial appearance is generally accepted as a common symptom. Along with a wide nasal bridge, an abnormal distance between the normally symmetrical paired organs, known as hypertelorism, is seen. In cases of malformations, the increase in height and weight stops after a certain age, and growth retardation is observed [1,2,13].

### 3.3. Recurrent Respiratory Tract Infections

Generally, clinical findings include recurrent upper respiratory tract infections, pulmonary emphysema, difficulty breathing during sleep, and otitis media. High sweat electrolytes and recurrent respiratory tract infections are also seen in cystic fibrosis. Fucose residues accumulate due to enzyme deficiency seen in fucosidosis, and sialic acid affects the viscoelasticity of mucus. Although there are connections between fucosidosis and cystic fibrosis, such as mucus-secreting ciliated epithelial areas and recurrent infections, the immune systems of fucosidosis are not directly affected, so this symptom can be eliminated by mucus cleaning in fucosidosis [1,26].

### 3.4. Dysostosis Multiplex

Some patients with fucosidosis have abnormalities in ossification. Bone anomalies called dysostosis multiplex include cervical platyspondylosis, wide ribs or deformities, scoliosis and hunchback deformity of the lumbar vertebrae, and odontoid processes [1,34].

### 3.5. Dermatological Abnormalities

The symptoms of fucosidosis include abnormalities on the skin. Skin lesions are usually seen as red or purple raised dots on the lower abdomen and genital area. Angiokeratoma corporis diffusum is more likely to be seen on the skin, depending on age, and although not pathognomonic, it raises suspicion of the diagnosis of fucosidosis. Angiokeratomas, which cover the whole body, are punctate deposits of capillaries within the papillary dermis [1,34]. Angiokeratomas are more likely to be seen in patients over 20 years of age than in patients under 10 years of age. The cause of this skin abnormality is unclear, but one view is that it is a condition in which undegradable glycolipids and glycoproteins accumulate in endothelial cells, leading to apoptosis of these cells and subsequent regeneration of the cells, resulting in capillaries [27]. Telangiectasias are known as permanent capillary dilations, and some patients have only telangiectasias without angiokeratomas. In addition to these skin conditions seen in fucosidosis, skin thickness, acrocyanosis, keratinocyte differentiation, hyperhidrosis, hypohidrosis, and transverse nail bands are also seen [1].

### 3.6. Organomegaly

Organomegaly, known as abnormal growth of internal organs in fucosidosis disease, has been recorded in some cases of fucosidosis disease. The most common of these and non-progressive is hepatospenomegaly, where both the liver and spleen grow simultaneously. Liver balloon or pseudogargoyle cells give a clear and swollen appearance as a result of periodic acid–Schiff stain analysis. At the same time, hepatic cells containing stored ceramide tetra and pentahexoid are observed in liver biopsies [1,28,29].

### 3.7. Ophthalmological Abnormalities

Although severe visual impairment is rarely seen in fucosidosis, it has been shown that storage material accumulates in conjunctival, retinal, and skin vessels. In addition, histological evaluation of conjunctival endothelial cells revealed clear vacuoles with a reticular structure similar to those seen in mucopolysaccharidoses and dark inclusions with dense granular material. In addition, mild corneal clouding, strabismus, and congestive papilledema have been reported in some cases [1].

### 3.8. Clinical Features

Fucosidosis is divided into two types according to the age of onset and clinical features. However, since fucosidosis is a rare disease, the diagnosis of patients described so far has been delayed and the clinical findings have varied, and the current view is that the severity of the clinical disorder is a single disorder with a wide range of symptoms rather than two distinct types [10,27]. Clinical symptoms of type I fucosidosis begin early (approximately 6 months of age). Neurological deterioration progresses rapidly, and patients usually die between the ages of 5 and 10. Affected individuals exhibit profound psychomotor retardation, seizures, coarse facial features, hepatosplenomegaly, skeletal abnormalities, and significant neurological deterioration. Type II progresses more slowly and, therefore, symptoms appear before the age of 2. Although patients rarely survive to the age of 30, they usually die in the second decade of life. The slower progression of neurological deterioration and the milder course of the disease have been considered features that distinguish it from type I. Psychomotor retardation is present but less severe, seizures are less frequent, and the coarse facial and skeletal features are milder. Neurological symptoms progress more slowly, allowing for some retention of motor and cognitive functions. In addition, the fact that angiokeratomas usually occur with age and that type II patients are more likely to survive into adulthood has led to the symptoms of angiokeratomas being suggestive of type II [1,10,27]. These differences (Table 2) in clinical presentation have significant implications for diagnosis and treatment. In type I fucosidosis, the rapid progression of symptoms often prompts early diagnosis in infancy through clinical examination, enzyme activity assays, or genetic testing to confirm *FUCA1* mutations. In type II, the milder progression can delay diagnosis, highlighting the need for careful monitoring of symptoms in young children. Treatment for both types primarily focuses on symptomatic management and supportive care, including seizure control, physical and occupational therapy, and addressing complications, like respiratory infections or mobility issues. For type I, the focus is on managing severe neurological symptoms, while in type II, the slower progression allows for more proactive management of developmental delays [9]

In the review conducted by Willems et al. in 1991 on 77 patients reported in 18 different countries, 95% mental disorder, 87% motor disorder, 78% growth retardation, 79% coarse facial appearance, 58% dysostosis multiplex, 78% recurrent infections, 52% angiokeratoma, 38% seizures, and 44% organomegaly were recorded [2]. In 2019, Wali et al. identified 89 cases of fucosidosis reported from 26 different countries/ethnic origins since Willems et al.’s review in their case report and literature review. Of these, 75 patients with sufficient clinical data were taken as the basis to determine the clinical spectrum. According to the results, 50–60% mental disorder, 50–60% motor disorder, 30–40% growth retardation, 60–70% coarse facial appearance, 50–60% dysostosis multiplex, 30–40% recurrent infections, 40–50% angiokeratoma, 10–20% seizures, and 30–40% organomegaly were observed [36]. When the clinical finding percentages of these two studies were examined, growth retardation, especially together with mental and motor disorders, was considered to be one of the important findings of the disease in fucosidosis. However, it was suggested that the inconsistencies observed in the results of both reports could be affected by inadequate or unsystematic collection of data or improved supportive treatments. In 2022, a patient with a new homozygous pathogenic variant and atypical clinical findings was described in a case report by Şanlı and Uysal, and in addition to this case, they summarized the clinical and molecular features of 13 Turkish patients reported and presented in the literature. In the clinical findings of the Turkish patients, 100% mental disorder, 100% motor disorder, 90% growth retardation, 100% coarse facial appearance, 54% dysostosis multiplex, 63% recurrent infections, 54% angiokeratoma, 27% seizures, and 27% organomegaly were recorded, and the findings of this study were found to be more consistent with the report by Willems et al. [13].

## 4. Molecular Perspective

The *FUCA1* gene is located on the short arm (p) of chromosome 1. It contains eight exons spanning 23 kb and encodes an α-L-fucosidase of 461 amino acids, of which 22 amino acids are the signal peptide and 439 amino acids are the mature protein [1,37]. The enzyme is a homotetramer of approximately 50–60 kDa that hydrolyzes fucosyl bonds by acting on natural oligosaccharide and glycosphingolipid substrates, resulting from changes in N-glycosylation and proteolytic processing [2].

Homozygous or heterozygous mutations occurring in the germline of the *FUCA1* gene cause α-L-fucosidase deficiency. In the deficiency of the enzyme, fucose-containing glycolipids and glycoproteins accumulate in various tissues and urine as a result of incomplete degradation of *N*- and *O*-glycosylproteins [37]. In the HGMD (Human Gene Mutation Database), for fucosidosis, a rare lysosomal storage disease transmitted as an autosomal recessive trait, 36 biallelic pathogenic variants have been reported so far. Six missense and eleven nonsense substitutions, along with eight small and five large deletions have been described. In addition, only three splice site variants have been described, including one small deletion, one complete deletion, and one stop-loss mutation. At the same time, since the genotype–phenotype correlation is not well defined, there is significant clinical variability in symptoms [1]. The genetic variability within the *FUCA1* gene significantly contributes to the phenotypic variability observed in fucosidosis patients. This study identified four novel mutations in Tunisian patients, including frameshift mutations (*p.K57Sfs*75*, *p.F77Sfs*55*), a missense mutation (*p.G332E*), and a splice-site mutation (*c.662+5g>c*). Frameshift mutations were associated with severe phenotypes (type I), characterized by early onset and rapid neurological deterioration due to the complete loss of enzymatic activity. In contrast, the compound heterozygous combination of *p.G332E* and *c.662+5g>c* in one patient resulted in a milder phenotype (type II), likely due to residual enzymatic activity. Structural modeling revealed that the missense mutation *p.G332E* disrupts protein stability near the catalytic site, impairing function. These findings highlight the strong genotype–phenotype correlation in fucosidosis and emphasize the impact of specific mutations on disease severity [38].

## 5. Diagnostic Criteria and Biomarkers

Clinical symptoms seen in fucosidosis may be the first step for the suspicion of the disease. However, the common symptoms seen with other lysosomal storage diseases are insufficient for the definitive diagnosis of this disease. When looking at early-onset symptoms, developmental delay or psychomotor regression is noticed in the first year of life. Neurological abnormalities such as hypotonia, spasticity, seizures, and loss of acquired motor or cognitive skills are observed. Cognitive impairment and motor dysfunction worsen over time, causing loss of speech, vision, and walking ability [1,24,25]. When looking at somatic symptoms, the patient is seen to have coarse facial features, macroglossia, recurrent respiratory tract infections, and skeletal anomalies [1,2,13,31]. Angiokeratomas are more common in advanced stages but can sometimes help with early diagnosis [27]. When looking at radiological features, advancements in diagnostic imaging, particularly in magnetic resonance imaging techniques, have significantly improved the differentiation of fucosidosis from other neurometabolic disorders. The continuously changing signal intensity and hypomyelination in the globus pallidus seen in the neuroimaging results raise high suspicion for the diagnosis of fucosidosis [39]. In addition, the suspicion rate is higher in societies where the consanguinity rate of fucosidosis is high. The presence of a history of similar symptoms in siblings and close relatives makes early diagnosis valuable for this disease.

**Figure 2:**
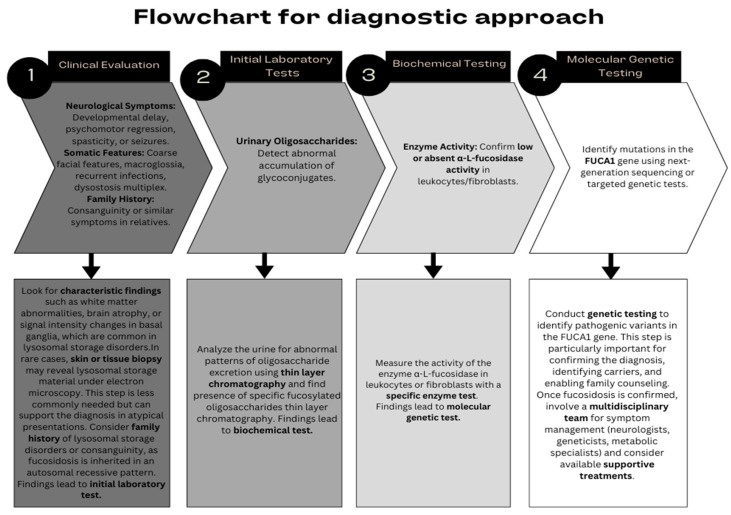
The flowchart for diagnostic approaches.

When looking at biomarkers for fucosidosis, brain magnetic resonance imaging findings in fucosidosis often reveal characteristic features such as progressive cerebral atrophy, white matter abnormalities, and signal hyperintensities on T2-weighted images, particularly in the periventricular regions and basal ganglia. Diffusion tensor imaging has also been used to detect microstructural changes in white matter, providing further specificity in identifying lysosomal storage disorders. These imaging patterns, combined with clinical and biochemical findings, allow for a more accurate differentiation of fucosidosis from similar conditions, such as metachromatic leukodystrophy or Krabbe disease [35]. In addition, the lack of α-L-fucosidase activity in patients’ leukocytes, fibroblasts, or plasma is a biomarker for fucosidosis. At the same time, excessive amounts of glycopeptides are seen in the urine of patients with fucosidosis. The presence of fucose-rich glycoconjugates in urine can be detected by thin-layer chromatography. However, the definitive diagnosis of the disease is proven by the analysis of mutations occurring in the *FUCA1* gene [40]. Individuals with distinct phenotypes can be diagnosed with *FUCA1* gene-targeted tests, but fucosidosis has a wide clinical presentation. Therefore, comprehensive tests, such as chromosomal microarray analysis or array comparative genomic hybridization (aCGH) analysis, are recommended for patients with inadequate clinical diagnosis. The clear application of these criteria (Figure 2) for early diagnosis, combined with increased awareness among healthcare professionals, can lead to earlier diagnoses and facilitate timely access to potential treatments such as hematopoietic stem cell transplantation, gene therapy, or enzyme replacement therapy before serious permanent damage occurs in patients. For this, prenatal diagnosis is important. Since fucosidosis is inherited as an autosomal recessive trait, biochemical tests are not recommended for prenatal diagnosis because mutation carriers still show partial protein expression. Therefore, if there is a family member affected by *FUCA1* pathogenic variants, prenatal testing or molecular genetic tests, such as preimplantation genetic testing (PGT-M), are used in subsequent pregnancies [1].

## 6. Treatment and Therapeutic Options

Since the first case of the disease was seen in 1966, studies have been carried out to define the disease, determine its clinical appearance, and examine it at the molecular level. However, due to the fact that it is a rare disease and few cases are seen or the cases that come are diagnosed with advanced disease and the disease progresses rapidly, studies on the treatment of the disease are slow. The treatment of fucosidosis is very difficult, and there is currently no definitive effective treatment. Therefore, symptomatic treatments are applied to improve the quality of life. The multidisciplinary team that applies these treatments consists of physiotherapists, orthopedists, cardiologists, ophthalmologists, and neurologists [13]. Since neurological symptoms and respiratory tract infections are the most important cause of early death in patients with fucosidosis, this problem needs to be treated early. Abnormally growing tissues that appear in the upper respiratory tract and protrude from the mucous membrane cause nasal polyps. This situation negatively affects the flow of passing air and causes problems such as nasal congestion, difficulty in smelling, and sleep apnea in the patient. Nasal polyps that do not go away on their own can be removed surgically. In the lower respiratory tract, albuterol, nebulized beclomethasone, and fluticasone inhalers can be used to relieve swelling and inflammation in the walls of the airways in the lungs. The airway clearance system is used to displace mucus in the bronchial walls from the small airways to the large airways to prevent atelectasis and pneumonia. Seizures, which are among the other symptoms, can be controlled with anti-epileptic treatment. However, involuntary contractions seen in patients with dystonia are prevented for a while with baclofen or by preventing the release of acetylcholine, a toxin produced by the *Colostridium botulinum* bacteria that carries the muscle contraction command. In addition to these treatments, physical massage therapy can also be applied [32].

Treatment of fucosidosis is a great challenge, and studies about effective treatments are still ongoing. Hematopoietic stem cell transplantation is promising as an effective method for the treatment of the rare disease fucosidosis after its early diagnosis. But, it has some disadvantages, such as the body’s immune system decreasing due to chemotherapy applied after transplantation, leaving the body vulnerable to microbes and infections, and the risk of death is high with this treatment [41]. In another treatment method, gene therapy, the use of retroviral vectors is promising due to their easy integration, high cell efficiency, and safety. However, effective delivery of gene therapy to the central nervous system remains a challenge. Strategies such as direct intrathecal administration or the use of vectors with central nervous system tropism have shown good results, but care must be taken to ensure that they are distributed equally across brain regions [42]. In another treatment approach, enzyme replacement therapy, preclinical studies are ongoing for fucosidosis, but the blood–brain barrier is a major obstacle in lysosomal storage diseases affecting the central nervous system. When applied early in the disease course and if the blood–brain barrier is overcome, it may offer better clinical outcomes by preventing the onset of irreversible damage [43].

### 6.1. Animal Models

#### 6.1.1. Canine

Fucosidosis due to deficiency of the α-L-fucosidase enzyme has been observed in Springer Spaniels in Australia and the United Kingdom. The mutant fucosidosis gene was transferred from England to Australia via dogs imported from English show clubs. Sick Springer Spaniels develop normally for 1 year and appear superior to healthy puppies. For this reason, they are frequently selected as show candidates. In 1971, an English Springer Spaniel was presented to Hartley and his team as a case study. Later, in 1978 and 1980, two dogs aged 2–3 years in Australia were observed to show progressive neuropathological signs. It was known that these two dogs were closely related to a dog previously imported from England to Australia. In a study published by Hartley et al. in 1982, they stated that these dogs had a loss of myelin and Purkinje cells in the central nervous system and that most of their neurons showed severe cytoplasmic vacuolization. They suggested that some of these vacuoles may contain oligosaccharide-like material [44]. In 1983, one of the puppies of these dogs was 12 months old and the other was 19 months old, and the same symptoms began to appear. The earliest clinical sign of canine fucosidosis is the failure to walk on the forelimbs with the hind legs held high. Later, tremors and numbness are observed, along with difficulty swallowing. Blindness, deafness, and progressive ataxia are among the symptoms of canine fucosidosis. Springer Spaniels die in a lethargic state between the ages of 3 and 5. Since canine fucosidosis is closely related to the symptoms of human fucosidosis, it is used as a potential animal model [45]. So far, the canine model has been used as a model for hematopoietic stem cells and enzyme replacement therapy treatments of fucosidosis. Consequently, the canine model has deepened our understanding of disease progression, particularly severe neurological involvement, and has shown the importance of early intervention to prevent irreversible damage. Studies in dogs have been pivotal in demonstrating the potential of intrathecal enzyme delivery to bypass the blood–brain barrier, offering hope for treating the neurological symptoms of fucosidosis [2].

#### 6.1.2. Mouse

For therapeutic research of fucosidosis treatment, the canine model has contributed greatly to the development of approaches, such as bone marrow transplantation and intracisternal enzyme replacement therapy, by crossing the blood–brain barrier. In order to easily establish more diagnostic and treatment strategies, a new knockout mouse model with symptoms similar to human fucosidosis was developed by Wolf et al. in 2016. While creating the knockout model, the *Escherichia coli* neomycin phosphotransferase I (nptI) gene was inserted into the exon 1 region of the *FUCA1* gene, which encodes lysosomal α-L-fucosidase. With analyses of enzyme levels and genomic and transcript studies, *FUCA1* deficiency in the mouse model was definitely confirmed, and it was shown that the mice were completely devoid of α-L-fucosidase activity in all tissue homogenates tested. As a result, storage vacuoles were observed in the internal organs of *FUCA1*-deficient mice and control littermates. Mice with normal weight and growth up to 6 months of age were indistinguishable from wild-type mice. Later, knockout mice showed impaired motor function. Mice subjected to motor and cognitive testing showed sensorimotor fragmentation, impaired spatial learning, and fear memory. To prevent unnecessary suffering of the animals due to the severe phenotype, knockout mice were euthanized at 9–11 months of age. This model has provided insights into the biochemical and cellular mechanisms underlying lysosomal dysfunction, particularly in neurons and glial cells. Subtle changes in sensorimotor and cognitive abilities to the status of neuropathology have been identified. Using molecular genetics and immunochemical techniques, lysosomal dysregulation and evidence of neuroinflammation and secondary storage of GM2 ganglioside have been shown [46,47].

This mouse model reflects the type II form of human fucosidosis, despite severe neuropathology. Unlike the canine model, although the mouse model shows histological changes in many regions of the central nervous system and contains storage materials, it lacks demyelination due to the loss of oligodendrocytes in the central nervous system [46]. However, the paucity of data on patient cases of the rare disease fucosidosis makes studies on the treatment of the disease difficult. Compared to the dog model, cost-reducing factors such as easier reproduction, a defined genetic background, shorter life span, and much lower body weight provide convenience. In addition, it has been shown that producing sufficient amounts of recombinant enzymes for high-dose and long-term treatments in mice is much more economical and feasible than the dog model.

### 6.2. Hematopoietic Cell Transplantation

Hematopoietic cell transplantation, such as bone marrow or umbilical cord blood transplantation, is a frequently used treatment method for lysosomal storage diseases. After transplantation, some cells that cross the blood–brain barrier can differentiate into microglia and secrete lysosomal enzymes [41].

Hematopoietic stem cell transplantation studies have been conducted on a fucosidosis canine model that best represents human fucosidosis. The first transplantation to fucosidosis Springer Spaniels was successfully performed by Taylor et al. The results showed that α-L-fucosidase enzyme activity returned to normal in many tissues and, especially, in neural tissues behind the blood–brain barrier. With this improvement in enzyme activity, improvement was observed in peripheral nerve and visceral lesions and central nervous system pathology. However, they emphasized that treatment together with early diagnosis of this disease, not after the onset of clinical symptoms, may be effective [48,49]. Hematopoietic stem cell transplantation was first performed in humans in 1995 on an 8-month-old boy by A Vellodi et al. Although his development was normal, an MRI scan was performed on the boy after symptoms of the disease were seen in his older brother. After the abnormalities were observed, a volunteer donor was found for stem cell transplantation. Although mild neurodevelopmental delay continued to be observed eighteen months after transplantation, his older brother showed much greater developmental delay at the same age [50]. When the 4-year follow-up period of the fucosidosis case first treated with bone marrow transplantation was examined, it was seen that the enzymatic activity gradually increased and psychomotor development improved, as observed in the MRI scan results [51]. In another study, a patient diagnosed with fucosidosis was treated with unrelated donor umbilical cord blood transplantation. After transplantation, the patient’s enzymatic activity was shown to return to normal, and his neurological symptoms improved. Umbilical cord blood transplantation is considered a new approach to treating fucosidosis patients who do not have a suitable bone marrow donor [25].

In conclusion, hematopoietic stem cell transplantation is promising as an effective method for the treatment of the rare disease fucosidosis after its early diagnosis. The progressive nature of fucosidosis means that irreversible damage, particularly in the central nervous system, accumulates over time. Before significant neurological or systemic damage occurs, identifying the disease early allows hematopoietic stem cell transplantation to be performed at a stage where it can effectively slow disease progression and prevent further accumulation of toxic substrates in lysosomes. The economic feasibility of this treatment method as a single intervention makes it an attractive option [52]. However, this treatment method is not effective when individuals are diagnosed with advanced disease. In addition, finding a suitable donor for bone marrow transplantation is difficult due to the presence of more than one child affected by fucosidosis in the family [36]. The body’s immune system is weakened due to chemotherapy applied after transplantation, leaving the body vulnerable to microbes and infections. For this reason, the risk of death is seen as high with this treatment method [41]. Gene therapy is also seen as one of the basic research methods in the future. However, hematopoietic stem cell transplantation treatment method studies are considered a treatment method that has reached the clinical stage to improve the quality of life of patients affected by fucosidosis [23].

### 6.3. Gene Therapy

In hematopoietic stem cell transplants, daughter cells that produce the missing enzyme can pass to the central nervous system, differentiate there, and secrete the lysosomal enzyme [53]. In another treatment method, gene therapy, hematopoietic stem cells are taken from the patient and genetically modified. The use of retroviral vectors for gene correction is gaining importance in genetic disease treatment studies because they contain complex transcriptional units and transfer stem cells with high efficiency. In addition, the integration of these vectors is safer than other vectors [54].

In a study, a full-length cDNA clone encoding the lysosomal hydrolase α-L-fucosidase was cloned into a retroviral vector. It was used to efficiently transfer the α-L-fucosidase gene into both human and canine fucosidosis fibroblasts in vitro. The studies resulted in the correction of the characteristic α-L-fucosidase enzyme deficiency [55]. Therefore, these retroviral constructs need to be developed using animal models for gene therapy studies of fucosidosis disease. Gene therapy is considered as one of the future research methods. Because gene therapy targets the root cause of fucosidosis by introducing a functional copy of the mutated *FUCA1* gene, it does not require repeated interventions compared to other treatment strategies. When applied early in the disease course, it may offer better clinical outcomes by preventing the onset of irreversible damage. However, effective delivery of gene therapy to the central nervous system remains a challenge. Strategies such as direct intrathecal administration or the use of vectors with central nervous system tropism have shown good results, but care must be taken to ensure that they are distributed equally across brain regions [42]. Regarding economic challenges, gene therapy is currently very expensive, which can limit access for patients, especially in countries with limited healthcare resources. Therefore, rare diseases, such as fucosidosis, often have limited funding for research and treatment development.

### 6.4. Enzyme Replacement Therapy

The use of enzymes as biopharmaceuticals continues to gain importance in biotechnological studies. Enzyme replacement has become a widely used therapy method, especially in lysosomal storage diseases where enzymatic activity is impaired or deficient [56]. Enzyme replacement therapy is a treatment method based on replacing an enzyme that is inadequately produced or absent in the body at regular intervals throughout life. Although its safety and efficacy were initially debated, it was developed as the first effective treatment for type I Gaucher disease by Brady et al. in the early 1990s. Later, this treatment method became available for other lysosomal storage diseases, such as Fabry disease, mucopolysaccharidosis type I, Pompe disease, and Niemann Pick B disease, and clinical studies gained momentum [57].

The enzymes to be used in this treatment method can be isolated from animals, plants, or microorganisms, but in order to improve tissue targeting, the closest forms of enzymes to mammals need to be determined. Therefore, recombinant production and characterization of modified, highly purified enzymes are important. Microbial-derived enzymes have advantages over plant and animal-derived enzymes due to their high catalytic activity, stability, ease of isolation, high yield, no undesirable by-product formation, and easy economic applicability. They are also important potential therapeutic agents due to their suitability for nanocarrier encapsulation, fusion proteins, PEGylation, and other genetic manipulation methods [58].

Enzyme replacement therapy is ongoing in preclinical studies for fucosidosis, but the blood–brain barrier is a major obstacle in lysosomal storage diseases affecting the central nervous system. In order to overcome this problem, for the first time in a study, dogs with fucosidosis were injected with the canine α-L-fucosidase intracisternal enzyme infusion method at regular intervals for 3 months starting from 8 weeks of age. After treatment, α-L-fucosidase reached all central nervous system regions, although it was found to be higher in areas close to the injection site (39–73% normal) and lower in deep brain structures (2.6–5.5% normal). The statistical results of this study showed that there was a large change in the affected enzyme-treated group animals compared to the affected vehicle-treated group animals. Additionally, it was observed that fucosyl-linked oligosaccharide accumulation was significantly reduced, especially in the spinal cord and brainstem, liver, bone marrow, and lymph nodes. At the same time, partial improvement in neuropathology was recorded. No antibody formation or inflammatory reaction was observed in plasma and cerebrospinal fluid after the injections. Although this treatment method is not completely curative at this dose and treatment frequency, it has been shown that the enzyme is well tolerated and safe when delivered directly to the central nervous system. It has also been concluded that early treatment reduces substrate storage [43]. Therefore, enzyme replacement therapy is promising for the treatment of fucosidosis [1]. In a study using a new knockout mouse model by Wolf et al. in 2016, an expression system was established for the production of human α-L-fucosidase in CHO-K1 cells. The purified enzyme was administered intravenously to mouse models. It was observed that the enzyme was effectively taken to the visceral organs. As a preliminary result, promising data were presented from the new knockout mouse model for therapeutic studies of human fucosidosis disease in the development of enzyme replacement therapy [59].

As a result, a significant challenge in enzyme replacement therapy for fucosidosis is the difficulty of delivering the therapeutic enzyme to the brain due to the protective blood–brain barrier, which hinders the treatment of the neurological symptoms central to the disease. Moreover, the enzyme is often cleared rapidly from the bloodstream, reducing its effectiveness in the body. In some cases, the immune system may recognize the replacement enzyme as foreign, potentially decreasing its efficiency and causing side effects. To overcome these challenges, future studies could aim to enhance the stability and targeting of the enzyme. Modifications to improve its delivery into lysosomes or attaching specific molecules to enable crossing the blood–brain barrier could be explored. Administering the enzyme directly into the cerebrospinal fluid, such as via intracerebroventricular or intracisternal routes, may bypass the blood–brain barrier and improve delivery to the brain. Additionally, combining enzyme replacement therapy with emerging therapies, like gene therapy or small-molecule chaperones, could provide a more comprehensive approach by addressing the underlying enzyme deficiency and improving residual enzyme function.

## 7. Conclusions

Fucosidosis is a rare lysosomal storage disease caused by α-L-fucosidase deficiency following a mutation in the *FUCA1* gene. In lysosomal storage disease, which is usually inherited as an autosomal recessive trait, sphingolipids, mucopolysaccharides, glycolipids, and oligosaccharides accumulate in the lysosomes of different tissues of the body. Lysosomal storage diseases are usually categorized according to the storage material that cannot be hydrolyzed. However, fucosidosis is named according to the non-hydrolyzable sugar, and both fucosylated glycolipids and fucosylated glycoproteins are stored in the lysosomes. Fucose-linked glycoconjugates negatively affect the activity of hydrolases by changing the substrate-binding properties of other hydrolases in the lysosomes or by affecting the stability of the enzymes. Therefore, in order for fucose-linked glycoconjugates to be separated into their monomers, fucose must first be removed. This dysregularity in lysosomes triggers oxidative stress, cell damage, and the subsequent onset of an inflammatory response. Dysfunction seen in lysosomes can lead to cell death. Especially, microglia, known as the protective cells of the brain, contain a large number of lysosomes in their cytoplasm. This dysfunction in lysosomes, which can cause cell death, greatly affects the central nervous system. Fucosidosis was initially divided into two types according to the age of onset and clinical features. However, since fucosidosis is a rare disease, the diagnosis of patients identified so far has been delayed and clinical findings have varied, and the current view is that the severity of the clinical disorder has been referred to as a single disorder with a wide range of symptoms rather than two separate types. Clinical symptoms of fucosidosis, called type I, begin early (approximately at 6 months of age). In type I, where neurological deterioration progresses rapidly, patients usually die between the ages of 5 and 10. Type II progresses more slowly and, therefore, symptoms appear before the age of 2. The slower progression of neurological deterioration and the mild course of the disease is considered to be a feature that distinguishes it from type I. Although patients rarely live to the age of 30, the majority die at an early age due to neurological deterioration or respiratory tract infections, usually in the second decade of their lives. In addition to the psychomotor regression and loss of mental functions seen in fucosidosis, symptoms such as growth retardation, coarse facial features, hepatosplenomegaly, telangiectasis or angiokeratomas, epilepsy, inguinal hernia, and dysostosis multiplex are also seen. The clinical symptoms seen in fucosidosis can be the first step for the suspicion of the disease, but the common symptoms seen with other lysosomal storage diseases are insufficient for the definitive diagnosis of this disease. The definitive diagnosis of the disease is proven by the analysis of mutations in the *FUCA1* gene. Although studies have been carried out since the first case of the disease was seen in 1966, such as defining the disease, determining the clinical appearance, and examining it at the molecular level, studies on the treatment of the disease have been slow due to the fact that it is a rare disease and that only a small number of cases are seen or that the cases that come are diagnosed with advanced disease or that the disease progresses rapidly. The treatment of fucosidosis is a great difficulty, and there is currently no definitive effective treatment. Therefore, symptomatic treatments are applied to improve the quality of life. Hematopoietic cell transplantation studies are continuing in the treatment of fucosidosis. The results of the study conducted on the canine model, which is a preserved form of the disease, showed that α-L-fucosidase enzyme activity returned to normal in many tissues, especially in the neural tissues behind the blood–brain barrier, and even with this improvement in enzyme activity, improvement was observed in peripheral nerves and visceral lesions and central nervous system pathology. However, they emphasized that it would be effective to diagnose this disease at an early age and apply treatment together with the onset of clinical symptoms. This method was first applied to humans in 1995 on an 8-month-old boy. When the 4-year follow-up period of the fucosidosis case treated for the first time with bone marrow transplantation is examined, it is seen that the enzymatic activity gradually increased and the psychomotor development improved, as observed in the MRI scan results. This treatment method promises to be an effective treatment method. The economic feasibility of this treatment method as a one-time intervention makes it an attractive option. However, this treatment method is not effective since individuals are diagnosed with advanced disease. In addition, since there is more than one child affected by fucosidosis in the family, finding a suitable donor for bone marrow transplantation makes this treatment method difficult. The body’s immune system decreases due to chemotherapy applied after transplantation, making the body open to microbes and infections. For this reason, the risk of death is seen as high in this treatment method. Nevertheless, hematopoietic stem cell transplantation treatment method studies are considered as a treatment method that has reached the clinical stage to improve the quality of life of patients affected by fucosidosis. In another treatment method, gene therapy, hematopoietic stem cells are taken from the patient and genetically modified. The use of retroviral vectors for gene correction is gaining importance in genetic disease treatment studies because they contain complex transcriptional units and transfer stem cells with high efficiency. In addition, the integration of these vectors is safer than other vectors. Therefore, these retroviral structures need to be developed using animal models in gene therapy studies of fucosidosis disease. Gene therapy is considered one of the research methods of the future. Another treatment approach, enzyme replacement therapy, is ongoing in preclinical studies for fucosidosis, but the blood–brain barrier is a major obstacle in lysosomal storage diseases affecting the central nervous system. In order to overcome this problem, in the first study, dogs with fucosidosis were injected with the enzyme at regular intervals for 3 months using the canine α-L-fucosidase intracisternal enzyme infusion method starting from the age of 8 weeks. Although this treatment method was not completely curative at this dose and treatment frequency, it showed that the enzyme delivered directly to the central nervous system was well tolerated and safe. It was also concluded that early treatment reduced substrate storage. Therefore, ERT is promising for the treatment of fucosidosis. But, the accessibility and feasibility of advanced therapies, such as hematopoietic stem cell transplantation, gene therapy, and enzyme replacement therapy, for rare diseases, like fucosidosis, are significantly influenced by socioeconomic factors. The high cost of these therapies poses a major barrier, particularly in low- and middle-income countries where healthcare resources are limited and insurance coverage may be inadequate. Additionally, the availability of specialized medical infrastructure and expertise required for these therapies is often concentrated in high-income regions, making access challenging for patients in underserved areas. Socioeconomic disparities can also impact early diagnosis and treatment initiation, as families with lower incomes may face obstacles such as delayed access to diagnostic tools, limited awareness, and logistical difficulties in reaching treatment centers. Furthermore, public health priorities in resource-limited settings often focus on more prevalent diseases, leaving rare diseases, like fucosidosis, underfunded and underserved. Cultural and educational factors may also influence the acceptance and understanding of these treatments, particularly in communities where consanguinity is common, potentially increasing the incidence of genetic disorders. As a conclusion, addressing these socioeconomic challenges requires global collaboration to reduce costs, increase funding for rare disease research, and improve the accessibility of advanced therapies through international aid and policy initiatives.

## Figures and Tables

**Table 1:** Clinical features of fucosidosis.

Symptoms	Clinical Features	References
Motor and mental dysfunction	Loss of walking ability, difficulty sitting and speaking, seizures, impairment of cognitive functions.	[1,24,25]
Facial and physical features	Coarse facies, wide nose bridge, growth retardation.	[1,2,13]
Recurrent respiratory infections	Emphysema, sleep apnea, loss of smell ability.	[1,26]
Radiologic abnormalities	Cervical platyspondyly, wide ribs or deformities, scoliosis and humpback deformity of the lumbar vertebrae, short odontoid processes.	[1]
Dermatological abnormalities	Angiokeratoma corporis diffusum, telenjiektaziler, skin thickness, acrocyanosis, keratinocyte differentiation, hyperhidrosis, hypohidrosis, and transverse nail bands.	[1,27]
Organomegaly	The most common and non-progressive is hepatospenomegaly, in which both the liver and spleen enlarge at the same time.	[1,28,29]
Ophthalmological abnormalities	Accumulation of storage material in conjunctival, retinal, and skin vessels, mild clouding of the corneas, strabismus, and congestive papilledema.	[1]

**Table 2:** Comparison of type I and type II fucosidosis [1,2,9,35].

Feature	Type I Fucosidosis	Type II Fucosidosis
Onset of symptoms	Early onset (usually within the first year of life)	Later onset (childhood to early adolescence)
Neurological symptoms	Severe and rapidly progressive	Milder and more slowly progressive
Psychomotor development	Significant delay and regression	Milder delays, some retained skills
Facial features	Coarse facial features develop early	Coarse facial features develop later
Skeletal abnormalities	Prominent dysostosis multiplex	Milder or less prominent dysostosis multiplex
Angiokeratomas	Typically absent in the early stages, may appear later	Often present
Organomegaly	Common (hepatosplenomegaly)	Less common or milder
Life expectancy	Typically reduced, with death often in early childhood	Longer, can survive into late childhood or adolescence
Seizures	Frequent and severe	Less frequent and milder
Motor skills	Severe spasticity, loss of ambulation	Mild to moderate spasticity, some mobility retained
Infections	Frequent respiratory infections	Less frequent infections
*FUCA1* activity	Severely deficient or absent	Reduced but detectable
Genetic mutations	More likely to have severe mutations (e.g., nonsense or frameshift)	Often milder mutations (e.g., missense)
